# The effect of oral motor activity on the athletic performance of professional golfers

**DOI:** 10.3389/fpsyg.2015.00750

**Published:** 2015-06-02

**Authors:** Steffen Ringhof, Daniel Hellmann, Florian Meier, Eike Etz, Hans J. Schindler, Thorsten Stein

**Affiliations:** ^1^BioMotion Center, Institute of Sports and Sports Science, Karlsruhe Institute of TechnologyKarlsruhe, Germany; ^2^Department of Prosthodontics, Dental School, University of HeidelbergHeidelberg, Germany; ^3^Research Group Biomechanics, Institute for Mechanics, Karlsruhe Institute of TechnologyKarlsruhe, Germany

**Keywords:** golf performance, drive, biting, jaw clenching, craniomandibular system

## Abstract

Human motor control is based on complex sensorimotor processes. Recent research has shown that neuromuscular activity of the craniomandibular system (CMS) might affect human motor control. In particular, improvements in postural stability and muscle strength have been observed as a result of voluntary jaw clenching. Potential benefits of jaw aligning appliances on muscle strength and golf performance have also been described. These reports are highly contradictory, however, and the oral motor task performed is often unclear. The purpose of our study was, therefore, to investigate the effect of submaximum biting on golf performance via shot precision and shot length over three different distances. Participants were 14 male professional golfers – seven with sleep bruxism and seven without – randomly performing golf shots over 60m, 160m, or driving distance while either biting on an oral splint or biting on their teeth; habitual jaw position served as the control condition. Statistical analysis revealed that oral motor activity did not systematically affect golf performance in respect of shot precision or shot length for 60m, 160 m, or driving distance. These findings were reinforced by impact variables such as club head speed and ball speed, which were also not indicative of significant effects. The results thus showed that the strength improvements and stabilizing effects described previously are, apparently, not transferable to such coordination-demanding sports as golf. This could be due to the divergent motor demands associated with postural control and muscle strength on the one hand and the complex coordination of a golf swing on the other. Interestingly, subjects without sleep bruxism performed significantly better at the short distance (60 m) than those with bruxism. Because of the multifactorial etiology of parafunctional CMS activity, conclusions about the need for dental treatment to improve sports performance are, however, completely unwarranted.

## Introduction

Human motor control is based on the complex interaction of dynamic processes comprising, e.g., diverse sensory systems, intermuscular and intramuscular synergy, and, thereby, coordination of several joints with several degrees of freedom (Horak, 2006).

In recent decades, numerous researches on human motor control have suggested the potential effect of dental occlusion and muscle activity of the craniomandibular system (CMS). These suggestions arose from animal studies which revealed neuroanatomical connection of the trigeminal nerve to several structures associated with postural control (Buisseret-Delmas et al., 1999). Trigeminal projections to all levels of the spinal cord have also been found (Ruggiero et al., 1981; Devoize et al., 2010). Subsequent investigation of the effects of oral motor activity among humans revealed modulation of reflexes (Miyahara et al., 1996) and facilitation of motor system excitability (Boroojerdi et al., 2000) as a result of jaw clenching. Takada et al. (2000) concluded that these effects might contribute to increased stability in stance rather than to smoothness of movements. Several studies have confirmed the neuromuscular effect of oral motor activity and different jaw relations on postural control during upright unperturbed stance (Bracco et al., 2004; Sforza et al., 2006; Sakaguchi et al., 2007; Tardieu et al., 2009). More precisely, decay of center of pressure displacements induced by submaximum biting has been revealed by posturographic analysis (Hellmann et al., 2011b; Ringhof et al., 2015). Similar to the stabilizing effects, significant increases in force production and rate of force development when clenching the jaw have been described (Forgione et al., 1991; Hiroshi, 2003; Ebben et al., 2008). Ebben et al. (2008) suggested that the effects were caused by concurrent activation potentiation which, in turn, enhanced the neural drive.

Increasing attention has also been focused on athletic performance and the potential benefits of oral appliances, in general, and mandibular orthopedic repositioning appliances (MORA), in particular. These devices were used either to voluntarily interfere with dental occlusion, and thus to disturb optimum systemic function, or to properly align the mandible relative to the maxilla, to achieve an effective physiologic state. In an experiment with highly proficient marksmen, performance was found to be significantly better when the mandible was in symmetric centric relation, as compared with intercuspal or lateral occlusion, an effect primarily attributed to postural stabilization (Gangloff et al., 2000). Ergogenic effects resulting from use of jaw-aligning appliances have also been observed in measurement of muscle strength (Kaufman and Kaufman, 1985; Forgione et al., 1991, 1992; Gelb et al., 1996; Arent et al., 2010; Dunn-Lewis et al., 2012). Significant increases in muscle strength of the upper and lower extremities and improvements in vertical jump height have been observed for athletes wearing oral devices. Some of this work has been criticized, however, primarily because of weak experimental design and lack of control conditions (Jakush, 1982; McArdle et al., 1984). Other studies, in turn, have failed to observe alteration of muscle strength as a result of the use of oral appliances (Cetin et al., 2009; Allen et al., 2014; Golem and Arent, 2015), thus questioning the aforementioned ergogenic effects. These results are further reinforced by studies using double-blind tests which claimed that performance enhancements by use of MORAs and other stabilizing splints are simply a result of placebo effects (Burkett and Bernstein, 1983; Allen et al., 1984; McArdle et al., 1984; Chiodo and Rosenstein, 1986).

Despite this controversy, many authors still argue in favor of performance benefits, and have examined further the effects of oral appliances in diverse sports. In this context, recent studies investigated the performance of golf professionals while using stabilizing splints. Whereas Egret et al. (2002) observed significant reductions in ball speed variability but no changes in average ball speed and kinematic pattern of the golf swing, Kwon et al. (2010) and Pae et al. (2013) observed significant improvements in driving distance and club head speed when the oral appliances were being used. Because accurate hitting of the ball and transfer of as much momentum as possible to the ball are important aspects of improving one’s driving distance (Hume et al., 2005), it was suggested that the improvements were induced by increased focus of attention at the moment of impact and/or increased muscle strength in the upper and lower extremities. The latest study by Pae et al. (2013) demonstrated, however, that use of an adjusted oral splint may aid optimization of driving distance and club head speed but not initial ball speed and putting accuracy. Improved driving distance hence seemed more likely to be the result of enhanced muscle strength rather than increased focus.

Some weak points of the abovementioned studies – which also might have contributed to the controversy – are the lack of information concerning the generated bite forces and the mandibular positions during the experiments. In particular, when assessing the impact of jaw-aligning appliances on strength and golf performance, the actual oral motor activity while wearing the splints mostly remained unknown (Allen et al., 2014). Other studies used simple over-the-counter appliances, which in turn altered jaw relation to an undefined position or irritated the subjects because of their uncomfortable fit (Golem and Arent, 2015). In the case that custom-made splints were applied, terms like centric relation were used to describe the experimental jaw position (Gangloff et al., 2000). But, since there is no international consensus about the definition of a physiological centric jaw relation (Keshvad and Winstanley, 2000), the common used phrase of symmetric positioning of the mandible in centric relation is not meaningful, and the jaw positions as experimental conditions are thus not comparable.

Because of the consistent effects of jaw clenching on motor system excitability, therefore, two important questions arise: first, are the contradictory reports merely a consequence of diverse, potentially affecting task instructions – i.e., to perform normally or to bite on the respective splint – and, second, does biting on oral devices lead to different results from biting on one’s teeth? This is of particular interest, because investigation of the effect of jaw clenching itself has not yet been reported for golf or similar coordination-demanding sports.

The purpose of this study was, therefore, to investigate the effect of controlled oral motor activity, in the form of submaximum biting tasks, on the athletic performance of professional golf players. Golf performance was evaluated for short (60 m), medium (160 m), and driving distances, and compared for three biting tasks – submaximum biting on one’s teeth, submaximum biting on an oral splint, and habitual jaw position (HJP), which served as the control condition. It was hypothesized that submaximum biting increases driving distance in general and, more specifically, biting on an oral splint improves driving distance to a greater extent than biting on one’s teeth. For 60 m and 160 m, however, the authors supposed that the shot precision is not affected by oral motor tasks.

## Materials and Methods

### Subjects

Fourteen professional golfers participated in this study. Subjects were exclusively male, all playing in the first or second German Golf League. The participants were naïve to the experimental procedure and had no known muscular or neurological diseases that could have affected their ability to perform the experiments. All the subjects had normal vision and presented with full dentition (except for third molars) in neutral occlusion (Angle class I). Moreover, they all had no symptoms of TMD (Reissmann et al., 2009), whereas seven reported sleep bruxism.

The study was reviewed and approved by the Ethics Committee of the German Sports University, Cologne (no. 38/12). All subjects gave their written informed consent to the experiments, which were conducted in accordance with the Declaration of Helsinki.

### Experimental Design

The effects of oral motor tasks on golf performance were assessed by use of a crossover design in which three different shot distances and three oral motor tasks were compared. All subjects completed five trials per shot distance per oral motor task, i.e., 45 golf shots in total. To avoid any effects of learning or fatigue, shot distances, and oral motor tasks were randomly assigned for each subject.

Before testing, each subject was given standardized verbal instructions about the experimental procedure. During a warm-up session subjects were familiarized with the golf shots and oral motor tasks, first separately and then in combination. This was to ensure the subjects were capable of constantly maintaining the respective jaw motor task at the desired activity level. Finally, maximum voluntary contraction (MVC) of the M. masseter was recorded.

#### Golf Shots

Golf shots were performed over three distances – short (60 m), medium (160 m), and driving distance (Drive). The required shot directions and lengths were displayed to the participants in the form of pylons which were positioned at the respective locations. Based on their individual capabilities, subjects chose a sand or lob wedge for 60 m, a ‘mid iron’ from five to seven for 160 m, and a driver for Drive, respectively. The subjects, however, were not allowed to change the clubs between shots over the same distance.

To quantify golf performance for all three shot distances, a radar-based system (TrackMan Pro; TrackMan A/S, Vedbæk, Denmark) was used. Trackman Pro is a commercially available product widely used by professional golfers and coaches. By tracking the club head and measuring the trajectory of the golf ball, TrackMan Pro delivers data on impact, ball flight characteristics as well as on shot distance and direction. With accuracy of 0.33 m at 100 m, this system thus provides appropriate and sufficiently precise information on golf performance.

#### Oral Motor Tasks

Before and during the golf swing, subjects were asked to bite either on their teeth (B_T_) or on an oral splint (B_S_); hitting with HJP served as the control condition. HJP in this context could, for instance, involve interocclusal spacing between mandible and maxilla or just biting activity as well.

B_T_ and B_S_ were both performed at submaximum masseter activity of 25% MVC. To control for this coordinative task, visual biofeedback of the electromyographic activity (EMG) of the masseter muscle was presented to the participants. The raw EMG signals were rectified, smoothed (100 points moving median), and scaled to the previously recorded MVC data in real time. The feedback monitor was directly positioned behind the golf ball, enabling the subjects to shift their gaze from the monitor to the ball without much head movement.

Electromyographic data for the masseter were recorded by use of bipolar surface electrodes (Ag/AgCl) and Noraxon telemetric equipment (TeleMyo 2400 G2; Noraxon, Scottsdale, AZ, USA). The EMG signals were collected with a sampling frequency of 1,000 Hz and amplified by a factor of 500. The electrodes, which had a diameter of 14 mm and a center-to-center distance of 20 mm, were applied bilaterally to the belly of the masseter, in line with the direction of the muscle fibers. The ground electrode was positioned on the seventh cervical vertebra. Before application, the skin over the participants’ muscles was properly prepared by shaving, abrasion, and cleaning with alcohol.

During B_S_ the subjects were asked to bite submaximally on an intra-oral splint. The splint used in the present study (Aqualizer, medium volume; Dentrade International, Cologne, Germany) was a commercially available device based on a fluid self-adjusting system which distributes bite force evenly across the bite. The splint thus enables a physiologic auto balanced static equilibrium of the CMS (Hellmann et al., 2011a) with an interocclusal vertical height of 1–3 mm.

All oral motor tasks had to be performed for at least three seconds before the golf shots. When this was achieved, the subjects were instructed to focus their attention on the golf shot, but to maintain the required activity level as best they could during the entire golf swing, as practiced during the warm-up session.

### Data Analysis

#### Golf Performance

To assess golf performance, diverse length-specific performance variables were included in the evaluation. With regard to the 60-m and 160-m shots, when golfers are seeking best approach to the pin, precision is the key factor determining golf performance. Hence, the resulting distance to pin (Pin_total_) was chosen as the dependent variable of interest. To give more detailed information on shot precision, both lateral (Pin_side_) and longitudinal (Pin_length_) distance to pin were also evaluated for each shot. The purpose of the Drive is, however, to transfer as much momentum as possible to the ball and thus achieve the desired shot length. Consequently, when investigating Drive performance, the shot length achieved (Carry) and Pin_side_ are of primary interest.

In addition to the abovementioned performance variables, club, and ball data were evaluated for all the shot distances tested. Impact variables included club head speed immediately before impact (Speed_club_) and ball speed immediately after impact (Speed_ball_). Moreover, the smash factor (Smash), represented as the ratio of Speed_ball_ to Speed_club_, and the launch angle (Angle), indicating the angle at which the ball takes off relative to the ground, were analyzed.

#### Masseter EMG

The masseter EMG signals not only served as biofeedback for the subjects, but were also assessed to investigate masseter activity before and during the golf swing. For this purpose, the raw signals were initially rectified, smoothed (100 ms), and scaled to the MVC data. These data were then used to compare intended (25% MVC) and actual masseter activity by calculating the average EMG values for the time the subjects remained in the address position (MA_pre_).

To moreover contrast masseter activity during the golf swing, the mean (MA_swing_) and maximum EMG amplitudes (MA_max_) from 900 ms before until 350 ms after impact with the ball were analyzed for the different test conditions. This time period corresponds to the mean duration of the swing of professional golfers, starting with initiation of the backswing and ending with the so-called follow-through (Egret et al., 2002; Meister et al., 2011).

### Statistics

Statistical analysis was performed by use of IBM SPSS Statistics 21.0 (IBM Corporation, Armonk, NY, USA). First, Kolmogorov–Smirnov tests were applied to confirm the normality of data distribution. Mauchly’s tests of sphericity were then conducted to determine whether the assumption of sphericity was violated. When this did occur, Greenhouse–Geisser estimates were used to correct for any violations.

To test for differences between subject characteristics (age, mass, height, body mass index, and handicap) of the bruxism and non-bruxism groups independent *t*-tests were conducted.

The effects of oral motor tasks (B_T_, B_S_, HJP) on golf performance were investigated by one-way repeated measures ANOVA, performed separately for each shot distance (60 m, 160 m, Drive). In a second step, two-way repeated measures ANOVA was conducted to test for statistical differences between subjects with and without sleep bruxism, and to reveal possible interaction effects with the oral motor tasks.

For EMG analysis, first, one-sample *t*-tests were used to contrast intended (25% MVC) and actual masseter activity before the golf swing (MA_pre_) for both submaximum biting tasks (B_T_, B_S_). Two-way repeated measures ANOVA, performed separately for each shot distance, was then conducted to detect statistical differences between MA_pre_ for oral motor tasks and for subjects with and without sleep bruxism. Finally, mean (MA_swing_) and maximum (MA_max_) masseter activity during the golf swing were compared by three-way repeated measures ANOVA in which sleep bruxism (Yes, No) acted as between-subject factor, and oral motor task (B_T_, B_S_, HJP) and shot distance (60 m, 160 m, Drive) served as within-subject factors. In each ANOVA, Bonferroni correction was used to adjust for multiple comparisons.

All results are reported as mean values with 95% confidence intervals. Partial eta squared ($\eta_{p}^{2}$) is indicated to give information about effect sizes. For small effects $\eta_{p}^{2}$ = 0.01, for medium effects $\eta_{p}^{2}$ = 0.06, and for large effects $\eta_{p}^{2}$ = 0.14 (Cohen, 1988; Richardson, 2011). For all statistical tests, the level of significance was set *a priori* to *p* = 0.05.

## Results

The subject characteristics are listed in **Table 1**. Independent *t*-tests indicated no significant differences between bruxism and non-bruxism groups in respect of the variables under investigation.

**Table 1:** Subject characteristics.

Group	Group size	Age	Height	Mass	BMI	HCP
	[*n*]	[years]	[*m*]	[kg]	[kg/m^2^]	
Total	14	21.39 ± 3.93	1.83 ± 0.04	74.43 ± 6.57	22.08 ± 1.37	0.1 ± 1.7
Bruxism	7	24.09 ± 5.18	1.85 ± 0.02	78.86 ± 7.19	22.93 ± 1.64	-1.5 ± 1.9
No bruxism	7	18.69 ± 0.94	1.81 ± 0.04	70.00 ± 5.14	21.23 ± 0.91	1.4 ± 1.0

BMI = body mass index; HCP = handicap.

### Golf Performance

**FIGURE 1:**
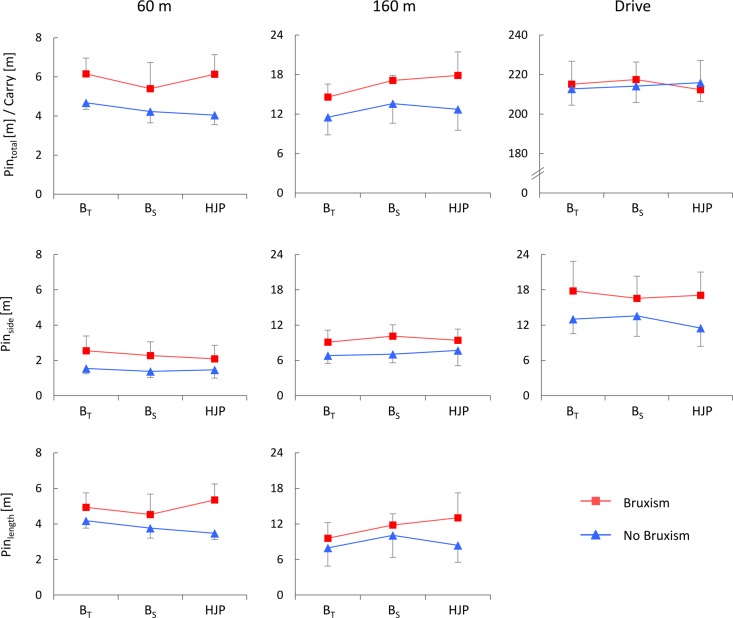
**Performance variables for 60, 160 m, and Drive distance as functions of oral motor tasks and sleep bruxism.** Pin_total_ = total distance to pin; Carry = shot length; Pin_side_ = lateral distance to pin; Pin_length_ = longitudinal distance to pin; B_T_ = biting on teeth; B_S_ = biting on splint; HJP = habitual jaw position.

**Figure 1** shows the length-specific performance variables as functions of the factors under investigation. Statistical analysis revealed that oral motor tasks did not statistically affect golf performance with respect to Pin_total_ for either the short (60 m) or medium distance (160 m). Apart from this, Pin_side_ was not significantly altered by the submaximum biting task, either for 60 or 160 m. These non-significant main effects of oral motor task were, moreover, found for Pin_length_ at 60 m. In contrast, oral motor tasks had a statistically significant effect on Pin_length_ at 160 m (*p* = 0.043, $\eta_{p}^{2}$ = 0.22). Bonferroni adjustments indicated that, compared with the golf shots under B_S_ and HJP, the distance from the pin was significantly reduced during B_T_ (*p* = 0.040 and *p* = 0.043, respectively). The submaximum biting tasks had no statistically significant effects on Carry at driving distance, however. There were, furthermore, no main effects on Pin_side_ at this distance. When subjects with and without sleep bruxism were compared, two-way repeated measures ANOVA revealed significant differences for Pin_total_ at 60 m (*p* = 0.035, $\eta_{p}^{2}$ = 0.32), indicative of better performance for subjects without bruxism, whereas for 160 m and Drive no main effects of sleep bruxism were observed. There were, in addition, no oral motor task × bruxism interaction effects for any performance variable. Detailed information on intra-individual and inter-individual performance as functions of the oral motor tasks – available in the supplementary material – shows that, particularly for the 60 m shot distance, some athletes (subjects 1, 4, 8, 9, 11, 13, and 14) benefited markedly from biting on the oral splint.

With regard to the impact variables (**Table 2**), oral motor tasks solely influenced Speed_club_ (*p* = 0.012, $\eta_{p}^{2}$ = 0.31) at 60 m. Bonferroni adjustments for multiple comparisons revealed significant differences between B_S_ and HJP (*p* = 0.014). For the 60 m shot distance, impact variables were also affected by sleep bruxism. Significant main effects were found for Speed_club_ (*p* = 0.032, $\eta_{p}^{2}$ = 0.33), Speed_ball_ (*p* = 0.003, $\eta_{p}^{2}$ = 0.53), and Angle (*p* = 0.049, $\eta_{p}^{2}$ = 0.29), whereas significantly higher speeds and larger angles were observed for subjects with bruxism. With regard to the 160 m shots, statistical analysis revealed significant differences exclusively for Smash (*p* = 0.005, $\eta_{p}^{2}$ = 0.50), in terms of higher factors for subjects without bruxism. Beside these effects, there were no interactions between oral motor tasks and sleep bruxism for any impact variable for all shot lengths, and no statistical differences related to Drive.

**Table 2:** Impact variables for each shot distance as functions of oral motor tasks and sleep bruxism.

	Bruxism			No bruxism		
	B_T_	B_S_	HJP	B_T_	B_S_	HJP
**Speed_**club**_ [m/s]**
60 m	28.89 ± 1.34	29.12 ± 1.40	28.54 ± 1.27	25.70 ± 1.38	26.09 ± 1.35	25.44 ± 1.60
160 m	41.46 ± 1.61	41.46 ± 1.54	41.55 ± 1.52	39.79 ± 1.04	39.78 ± 1.11	39.67 ± 1.02
Drive	48.23 ± 1.69	48.27 ± 1.64	48.15 ± 1.81	46.58 ± 1.51	46.67 ± 1.61	46.60 ± 1.58
**Speed_**ball**_ [m/s]**
60 m	29.15 ± 0.53	29.14 ± 0.51	29.07 ± 0.38	27.97 ± 0.46	27.98 ± 0.33	27.57 ± 0.50
160 m	54.82 ± 1.84	54.34 ± 1.20	53.98 ± 2.10	54.05 ± 1.09	54.05 ± 1.40	54.03 ± 1.03
Drive	68.45 ± 2.53	68.71 ± 2.48	68.09 ± 3.13	67.19 ± 2.00	67.07 ± 1.92	66.86 ± 2.21
**Smash [%]**
60 m	1.02 ± 0.05	1.01 ± 0.04	1.02 ± 0.04	1.10 ± 0.06	1.08 ± 0.06	1.10 ± 0.07
160 m	1.32 ± 0.02	1.31 ± 0.03	1.30 ± 0.02	1.36 ± 0.01	1.36 ± 0.01	1.36 ± 0.01
Drive	1.42 ± 0.03	1.42 ± 0.02	1.41 ± 0.03	1.44 ± 0.02	1.44 ± 0.02	1.44 ± 0.02
**Angle [∘]**
60 m	39.32 ± 1.83	39.59 ± 1.31	39.48 ± 1.36	36.34 ± 2.06	36.31 ± 2.18	36.11 ± 1.34
160 m	15.51 ± 1.14	14.96 ± 0.99	15.28 ± 1.00	13.57 ± 1.11	13.23 ± 1.00	13.67 ± 1.02
Drive	12.70 ± 1.34	12.49 ± 1.33	13.22 ± 1.64	12.09 ± 0.46	12.62 ± 0.63	12.87 ± 0.42

B_*T*_ = biting on teeth; B_*S*_ = biting on splint; HJP = habitual jaw position; Speed_*club*_ = club head speed immediately before impact with the ball; Speed_*ball*_ = ball speed immediately after impact of the club; Smash = smash factor (Speed_*ball*_/Speed_*club*_); Angle = angle at which the ball takes off, relative to the ground.

### Masseter EMG

**FIGURE 2:**
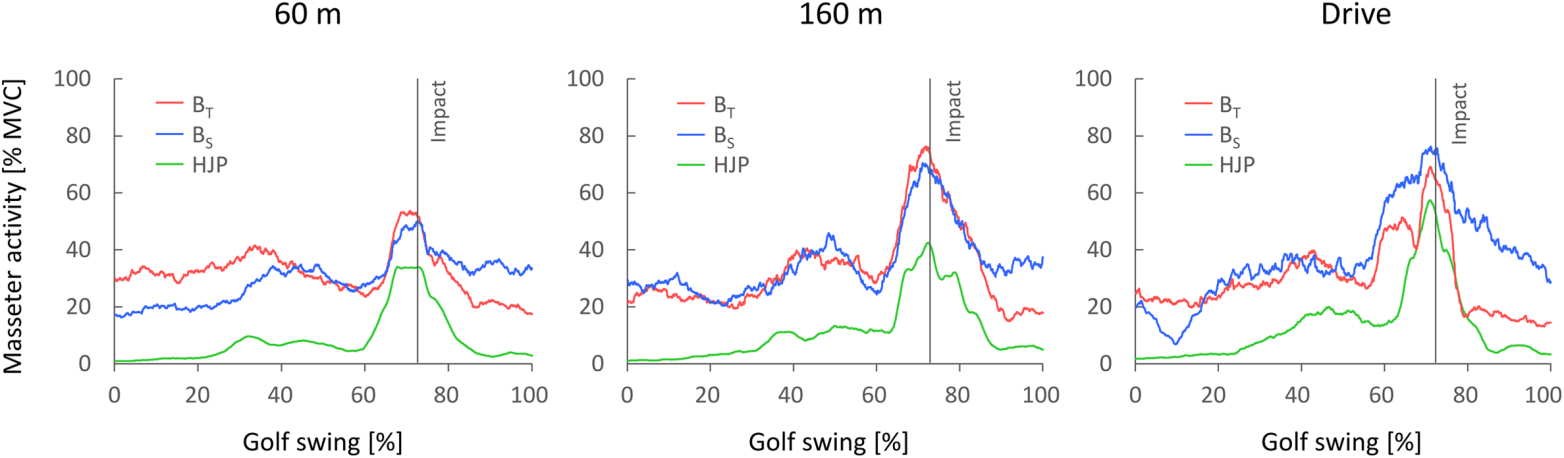
**Typical time courses of the masseter activity from initiation of the backswing (0%) until follow-through (100%) for the different test conditions.** BT = biting on teeth; BS = biting on splint; HJP = habitual jaw position.

All results relating to masseter activity before (MA_pre_) and during the golf swing (MA_swing_, MA_max_) are listed in **Table 3**. Typical time courses of the masseter activity during the golf swing can be obtained from **Figure 2**.

**Table 3:** Masseter activity before and during the golf swing for each shot distance as functions of oral motor tasks and sleep bruxism.

	Bruxism			No bruxism		
	B_T_	B_S_	HJP	B_T_	B_S_	HJP
**MA_**pre**_ [%]**
60 m	23.84 ± 1.79	23.99 ± 1.71	2.14 ± 0.74	24.81 ± 3.44	23.65 ± 3.72	1.98 ± 0.60
160 m	23.41 ± 1.26	24.37 ± 2.29	1.98 ± 0.74	23.42 ± 2.85	22.83 ± 3.80	2.77 ± 1.50
Drive	25.46 ± 2.29	25.11 ± 2.63	1.77 ± 0.83	24.61 ± 3.46	22.51 ± 3.43	1.84 ± 0.64
**MA_**swing**_ [%]**
60 m	17.10 ± 7.68	21.68 ± 5.65	10.30 ± 8.23	21.52 ± 6.35	19.63 ± 5.55	9.08 ± 6.51
160 m	23.85 ± 10.68	26.85 ± 8.58	17.35 ± 11.55	24.34 ± 7.43	28.26 ± 7.12	15.32 ± 10.37
Drive	26.33 ± 9.80	33.32 ± 10.87	18.45 ± 12.08	30.80 ± 7.75	33.03 ± 8.63	14.29 ± 7.17
**MA_**max**_ [%]**
60 m	43.11 ± 25.88	49.59 ± 19.23	38.25 ± 30.95	57.37 ± 32.83	50.95 ± 27.64	36.07 ± 29.58
160 m	71.56 ± 39.91	72.41 ± 35.17	69.34 ± 41.64	71.36 ± 32.09	77.40 ± 37.10	56.09 ± 37.15
Drive	79.09 ± 39.19	87.09 ± 42.11	67.30 ± 39.40	82.15 ± 33.23	80.36 ± 33.91	48.33 ± 20.91

B_*T*_ = biting on teeth; B_*S*_ = biting on splint; HJP = habitual jaw position; MA_*pre*_ = mean masseter activity before golf swing; MA_*swing*_ = mean masseter activity during golf swing; MA_*max*_ = maximum masseter activity during golf swing.

With regard to MA_pre_, one-sampled *t*-tests showed that for neither B_T_ nor B_S_ did the effectively realized masseter activity deviate significantly from the intended activity of 25% MVC. This was true for all shot distances and both subpopulations, i.e., subjects with and without sleep bruxism. Two-way repeated measures ANOVA revealed, moreover, no statistical difference between MA_pre_ for B_S_ and B_T_, but significant less masseter activity during HJP than during B_S_ and B_T_ for all shot distances (each *p* < 0.001).

For MA_swing_, three-way repeated measures ANOVA indicated main effects of oral motor task (*p* < 0.001, $\eta_{p}^{2}$ = 0.74) and shot distance (*p* < 0.001, $\eta_{p}^{2}$ = 0.66). MA_swing_ increased significantly with shot distance (60 m vs. 160 m: *p* = 0.007; 60 m vs. Drive: *p* = 0.001; 160 m vs. Drive: *p* = 0.013), and was significantly higher for B_T_ and B_S_ than for HJP (*p* = 0.001 and *p* < 0.001, respectively). There were, in contrast, no statistically significant differences between B_T_ and B_S_, and no bruxism or interaction effects.

Similar results were obtained for MA_max_. Statistical analysis revealed main effects of oral motor task (*p* < 0.001, $\eta_{p}^{2}$ = 0.60) and shot distance (*p* < 0.001, $\eta_{p}^{2}$ = 0.60). Bonferroni adjustments for multiple comparisons indicated that MA_max_ was significantly lower for HJP and 60 m than for the submaximum oral motor tasks (B_T_ vs. HJP: *p* = 0.002; B_S_ vs. HJP: *p* = 0.001) and the longer shot distances (60 m vs. 160 m: *p* = 0.005; 60 m vs. Drive: *p* = 0.002), respectively. In addition, a significant oral motor task × bruxism interaction effect was observed (*p* = 0.044, $\eta_{p}^{2}$ = 0.25). Whereas for subjects without sleep bruxism a clearly different MA_max_ between HJP and both submaximum biting tasks was observed, the EMG amplitudes for subjects with bruxism were very high during HJP and only slightly lower than those during B_T_ and B_S_.

## Discussion

The purpose of this study was to investigate the effects of oral motor activity on the athletic performance of professional golfers. The authors hypothesized that submaximum biting would significantly increase drive distance whereas shot precision at 60 and 160 m would be unaffected by these jaw motor tasks.

First, it must be mentioned that the requested activity level before the shot (25% MVC) was achieved by the subjects for both force-controlled biting conditions, with no statistical differences between B_S_ and B_T_, but significantly higher MA_pre_ than during HJP. This forms the basis for the further discussion, enabling comparability of the results and conclusive statements.

With regard to the primary length-specific performance variables (Pin_total_ at 60 and 160 m, and Carry at Drive), statistical analysis revealed no significant differences between oral motor activity. Even when golf performance is considered in more detail, neither lateral (Pin_side_) nor longitudinal (Pin_length_) distance to pin were statistically affected by submaximum biting. The only exception was for Pin_length_ at 160 m, which was significantly improved for B_T_ compared with B_S_ and HJP. Outcomes were similar for the impact variables (Speed_club_, Speed_ball_, Smash, Angle); again only Speed_club_ at 60 m changed as a result of the oral motor tasks, with the velocity of the club head during B_S_ being significantly higher than during HJP. These results thus showed that, under the study conditions chosen, biting at a submaximum level did not systematically improve golf performance with regard to shot precision and shot length over three different shot distances; this conclusion is reinforced by the absence of statistically significant differences for the impact variables. In this context, it should also be noted that biting on the splint used in our study did not affect golf performance differently from biting on one’s teeth.

To the best of our knowledge, this study was the first to examine the effect of submaximum biting on golf performance. The results cannot, therefore, be compared with those from previous studies. For this reason the authors focus on discussion of the general effect of the CMS on human movement in an attempt to provide possible explanations, without any claim to be comprehensive. As already indicated above, several reports have described potential performance benefits, particularly improvements in strength (Kaufman and Kaufman, 1985; Forgione et al., 1991; Gelb et al., 1996; Dunn-Lewis et al., 2012), and driving distance (Kwon et al., 2010; Pae et al., 2013), induced by the use of jaw-aligning appliances. Taking into account that driving distance is very dependent on club head speed, which is, in turn, closely related to muscle strength of the upper and lower extremities (Hellström, 2009), it has been hypothesized that increases in driving distance resulted from improvement of muscle strength, possibly as a result of optimum systemic function and reduced stress on the CMS, which is assumed to be important for achieving maximum athletic potential (Gabaree, 1981; Pae et al., 2013).

Ergogenic effects on muscle strength and power have also been described for jaw-clenching tasks. When the jaw was clenched, Hiroshi (2003) and Ebben et al. (2008) observed significant increases in peak force and rate of force development during grip strength assessments and countermovement jumps, respectively. The latter authors suggested that these improvements were provoked by concurrent activation potentiation, which increased the neural drive to the skeletal muscles, thus, gaining the athlete an ergogenic advantage during strength-related motor tasks (Ebben et al., 2008). In this study, however, submaximum biting tasks were not shown to significantly improve the participants’ driving distance or club head speed. There might be different reasons for this. First, the facilitating (Miyahara et al., 1996; Boroojerdi et al., 2000) and stabilizing (Gangloff et al., 2000; Hellmann et al., 2011b; Ringhof et al., 2015) effects of voluntary jaw clenching are not transferable to coordination-demanding full-body motion, like golf swings. This could be due to the divergent motor demands associated with postural control and shooting on the one hand, and golf swing on the other. Whereas the former are primarily based on feedback mechanisms and fine motor control (Horak, 2006), the latter requires whole-body coordination mainly associated with feedforward control – especially in experts. Modulation of somatosensory input, particularly for the neck muscles (Abrahams, 1977), and facilitation of ankle extensor and flexor muscles concomitant with attenuated reciprocal Ia inhibition from the pretibial muscles to the soleus muscle (Takada et al., 2000) by means of trigeminal connections and projections (Ruggiero et al., 1981; Devoize et al., 2010) might, thus, be not an issue. Second, golf swings are not just simple strength-related, single, or double joint movements. Golfers usually try to increase the torque applied to the club by summation of speed on the basis of successive actions of the hip, trunk, and shoulders, followed by motion of the arms, wrists, and hands (Burden et al., 1998; Egret et al., 2002). One must, therefore, question to what extent golf swings actually depend on muscle strength of the upper and lower extremities, and whether the observed performance benefits resulting from use of jaw-aligning appliances (Egret et al., 2002; Kwon et al., 2010; Pae et al., 2013) are effectively due to strength improvements. This investigation cannot resolve this question, however.

The last, and probably most conclusive, factor to be considered is that the abovementioned research on the impact of jaw clenching on muscular force development used an open mouth, non-clenching condition as control (Hiroshi, 2003; Ebben et al., 2008). Specifically, the participants in the investigations of Hiroshi (2003) and Ebben et al. (2008) were instructed to clench their jaw to the maximum extent or to keep their mouth open while performing the grip strength tests and countermovement jumps, respectively. In the present study, however, submaximum biting was compared with HJP, in which subjects were asked to perform the golf shots as normally as possible. In this context, it should be mentioned that, on the one hand, even professional golfers could not easily perform golf swings with their mouth open (Egret et al., 2002); on the other hand it should be noted that the subjects in our study, even under habitual conditions, clenched their teeth while performing the golf shots. Interestingly, both mean and maximum masseter activity during the golf swing increased significantly with requested shot distance. Clenching the jaw, hence, might be a common physiological strategy used to improve the neural drive to distal body segments and by this means enhance performance. This, in turn, would indicate that Hiroshi (2003) and Ebben et al. (2008) actually did not observe muscle strength improvements when the jaw was clenched, but rather a decrease in force development during the non-clenching condition.

Although the effect of bruxism was not the focus of this study, it had significant impact on golf shots over the short distance. Descriptively, all performance variables turned out worse for the golfers with sleep bruxism, especially under HJP conditions. Statistically, however, only Pin_total_ at 60 m was revealed to be significantly worse as compared to the healthy subjects, possibly as a result of greater club head speed and ball speed at impact. There is consensus about the multifactorial nature of the etiology of bruxism. In the past, morphological factors, for example occlusal discrepancies and the anatomy of the bony structures of the orofacial region, were believed to be the main causative factors of bruxism. Nowadays, however, these factors are believed to be of minor or no importance. It has been suggested that bruxism is part of a sleep arousal response modulated by a variety of neurotransmitters in the central nervous system. More specifically, disturbances in the central dopaminergic system have been linked to bruxism. Psychological factors, for example stress and personality, are also frequently mentioned in relation to bruxism, but research results are controversial (Lobbezoo and Naeije, 2000; Cuccia, 2008). Considering the multifactorial etiology of bruxism, further research is needed to elucidate the potential influence of bruxism on the performance of professional golfers. On the basis of our results it might be speculated that bruxism causes structural and functional changes (Ahlgren et al., 1969; Iida et al., 2014), which finally might impair motor performance during coordination-demanding tasks. The authors would like to point out, however, that on the basis of the present study and the literature available, conclusions on the need for dental treatment to improve sports performance are completely unwarranted.

## Conclusion

This study has demonstrated that jaw motor activity, in terms of submaximum biting, did not systematically affect the performance of professional golfers; whereas no differences were observed for biting on an oral splint, biting on one’s teeth, and HJP. On the other hand, it can be stated that neither submaximum biting nor the oral appliance used in our investigation impeded the athletes’ golf performance significantly. Essentially, however, particularly in high-level sports, the athlete and potential intervention to improve performance should always be regarded individually. Notwithstanding this, it remains unclear whether the contradictory reports regarding muscle strength and golf assessment in combination with jaw clenching or jaw aligning appliances are not just the result of divergent methods and control conditions. Future studies should, thus, contrast the effects of oral motor activities as a result of both open mouth and habitual conditions.

## Conflict of Interest Statement

The authors declare that the research was conducted in the absence of any commercial or financial relationships that could be construed as a potential conflict of interest.
