# Supplementary material for: The effect of oral motor activity on the athletic performance of professional golfers
Source: Front Psychol. 2015 Jun 2;6:750. doi: 10.3389/fpsyg.2015.00750 (PMC4451241; doi:10.3389/fpsyg.2015.00750)
Supplement: Supplementary file 1 [file Data_Sheet_1.PDF]

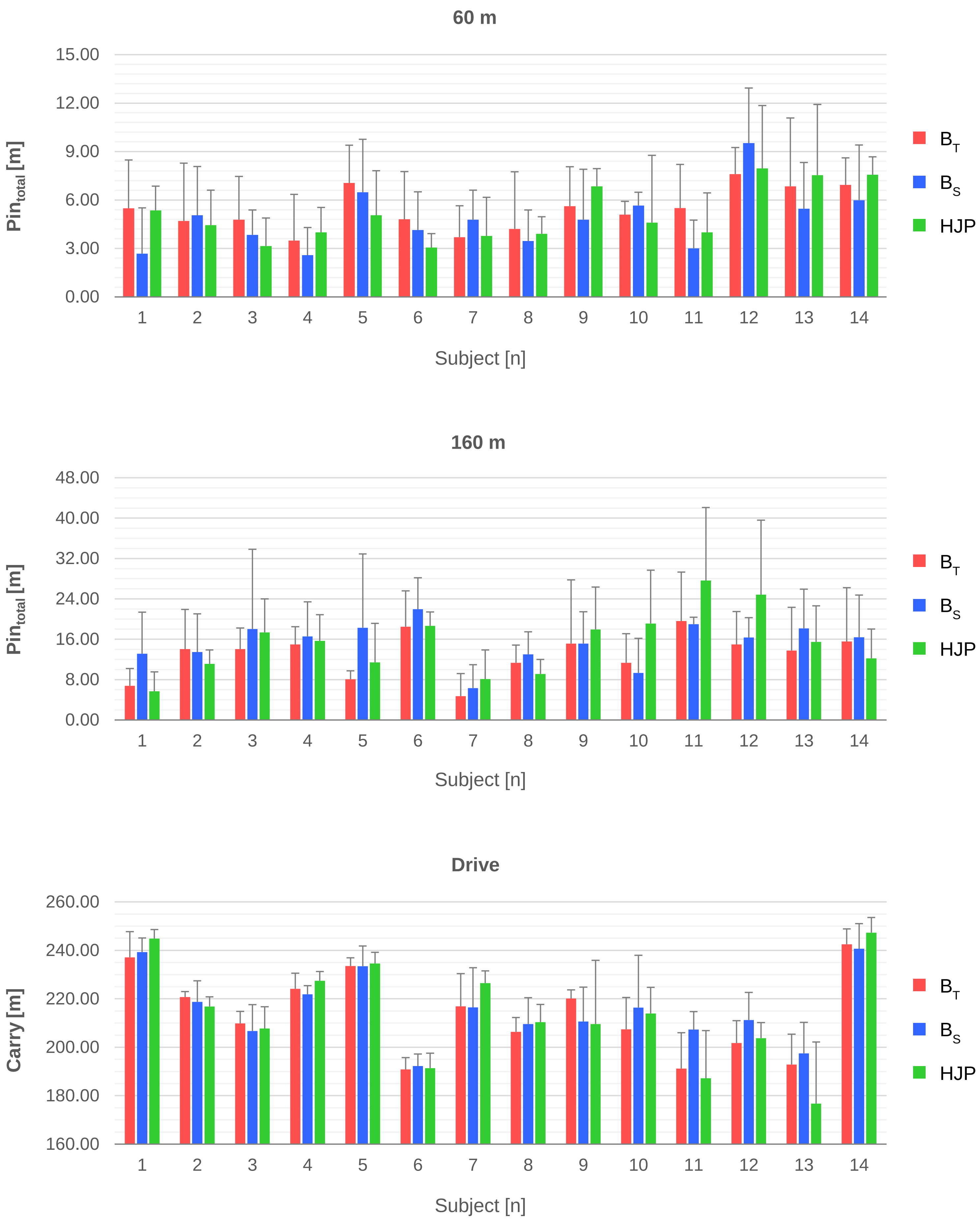

Figures Intra-individual und inter-individual comparisons of golf performance for 60 m, 160 m and Drive as functions of oral motor tasks, quantified by Pin_{total} and Carry, respectively.
Pin_{total} = total distance to pin; Carry = shot length; B_T = biting on teeth, B_S = biting on splint, HJP = habitual jaw position
